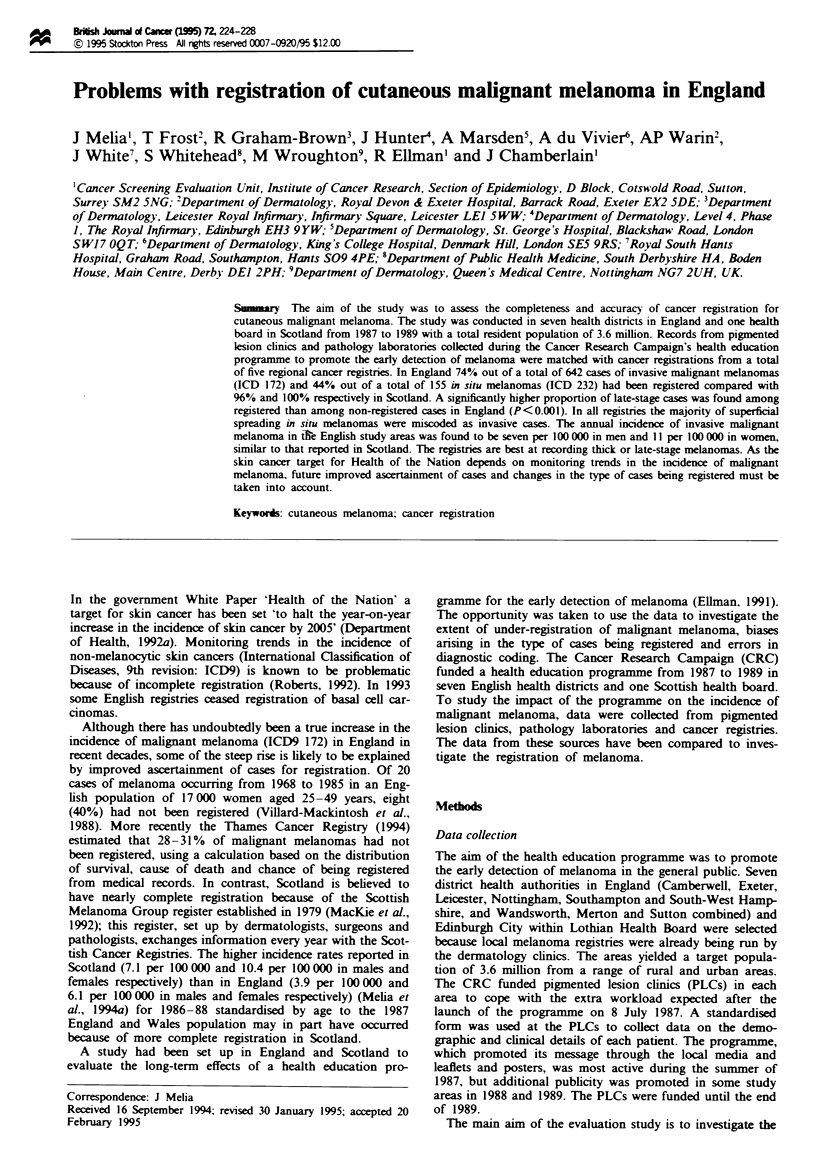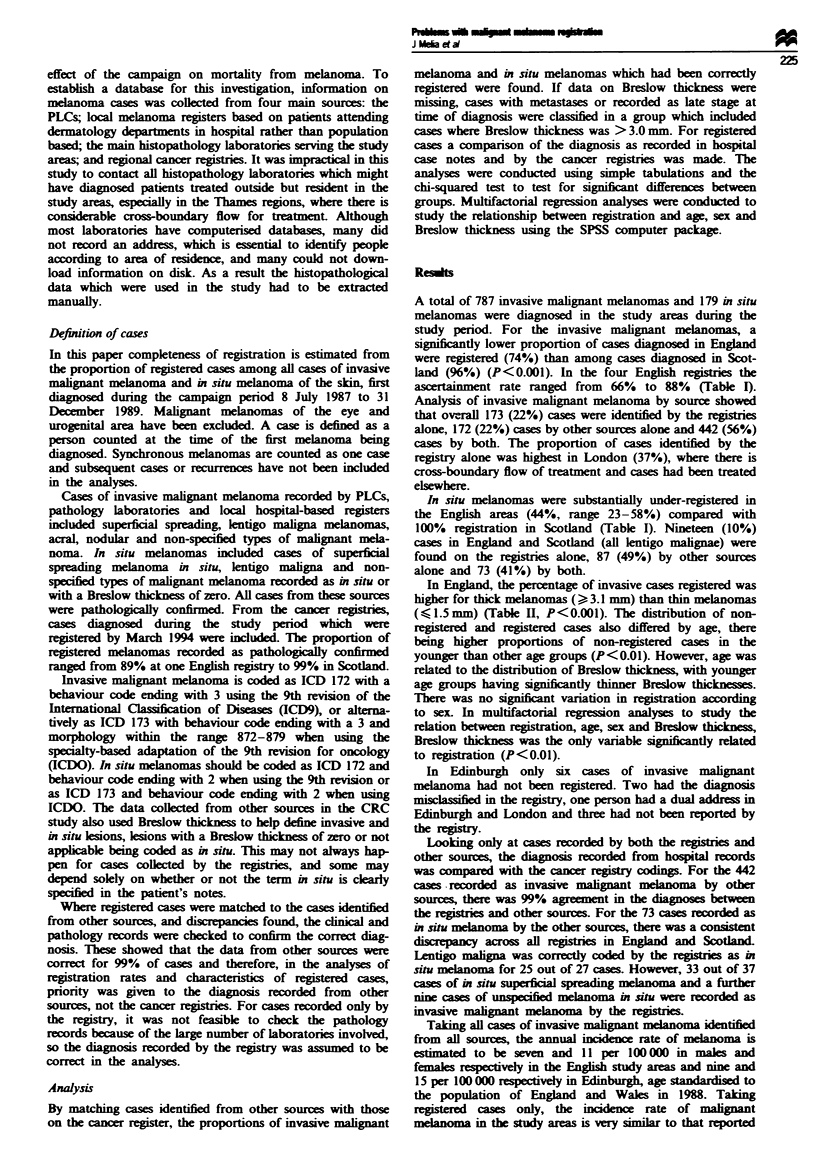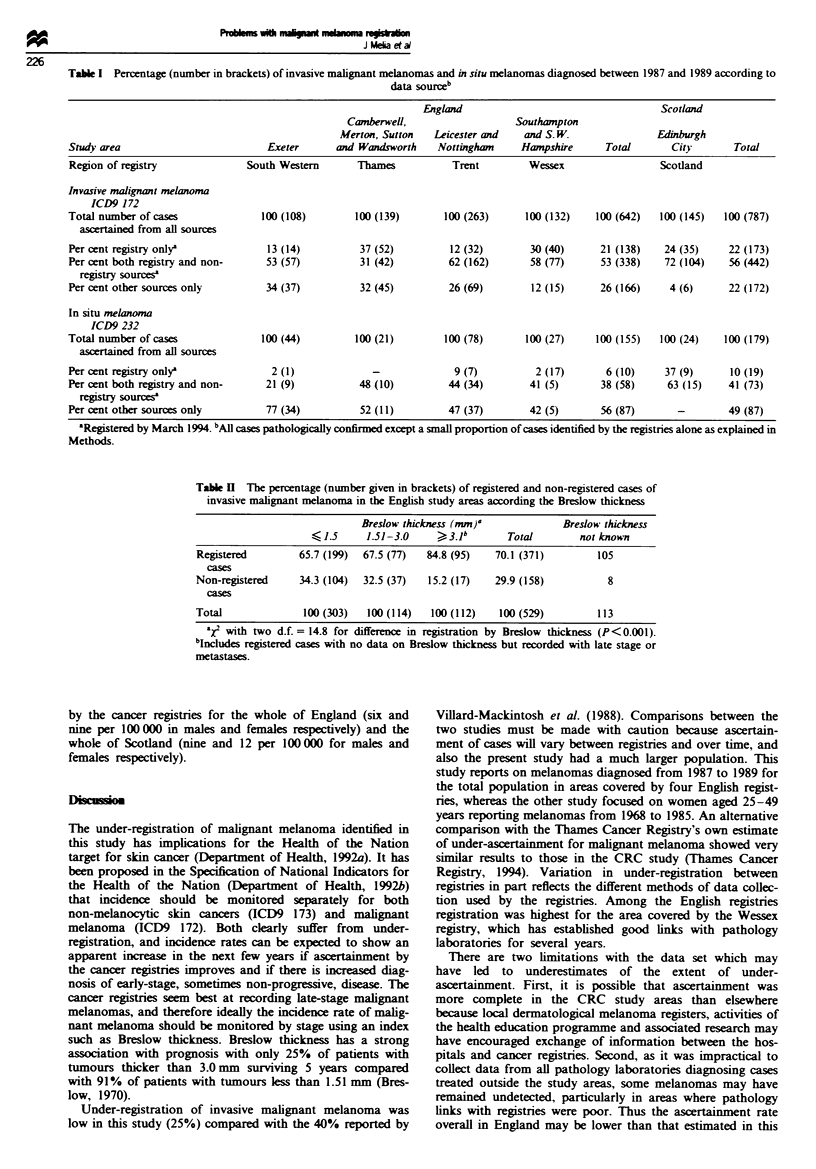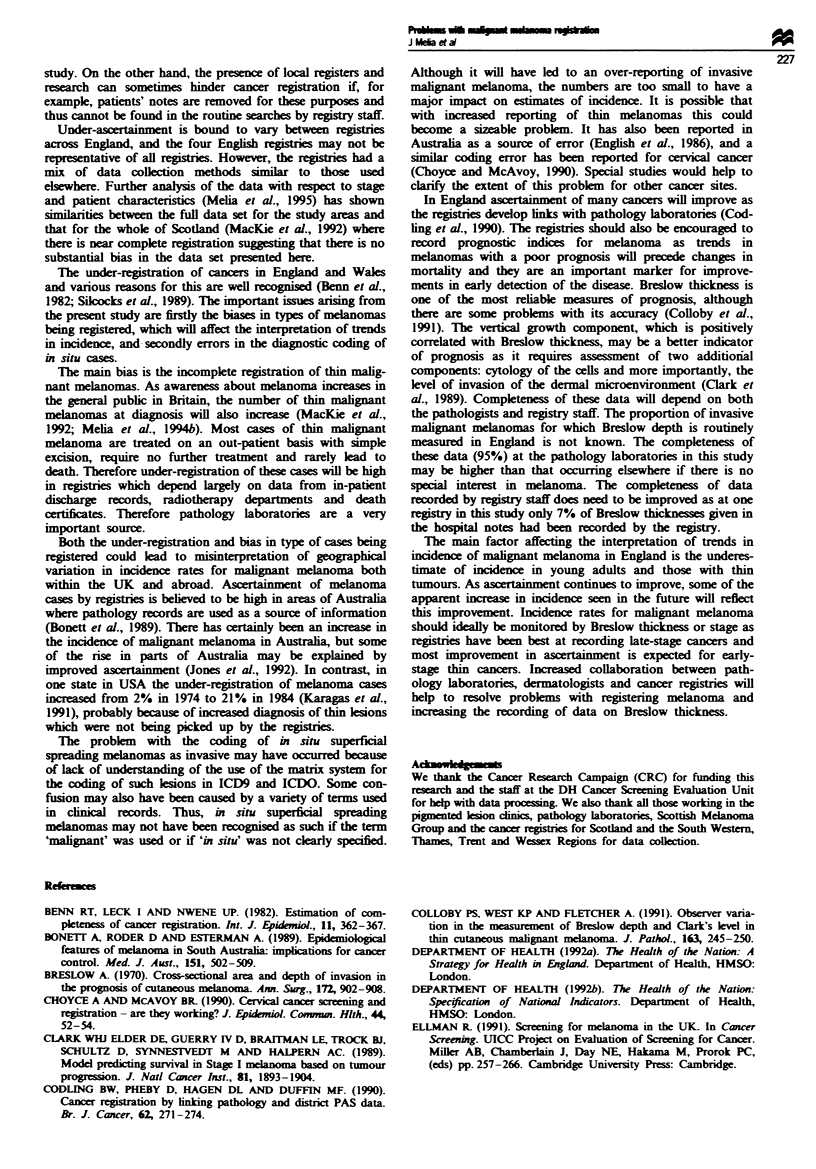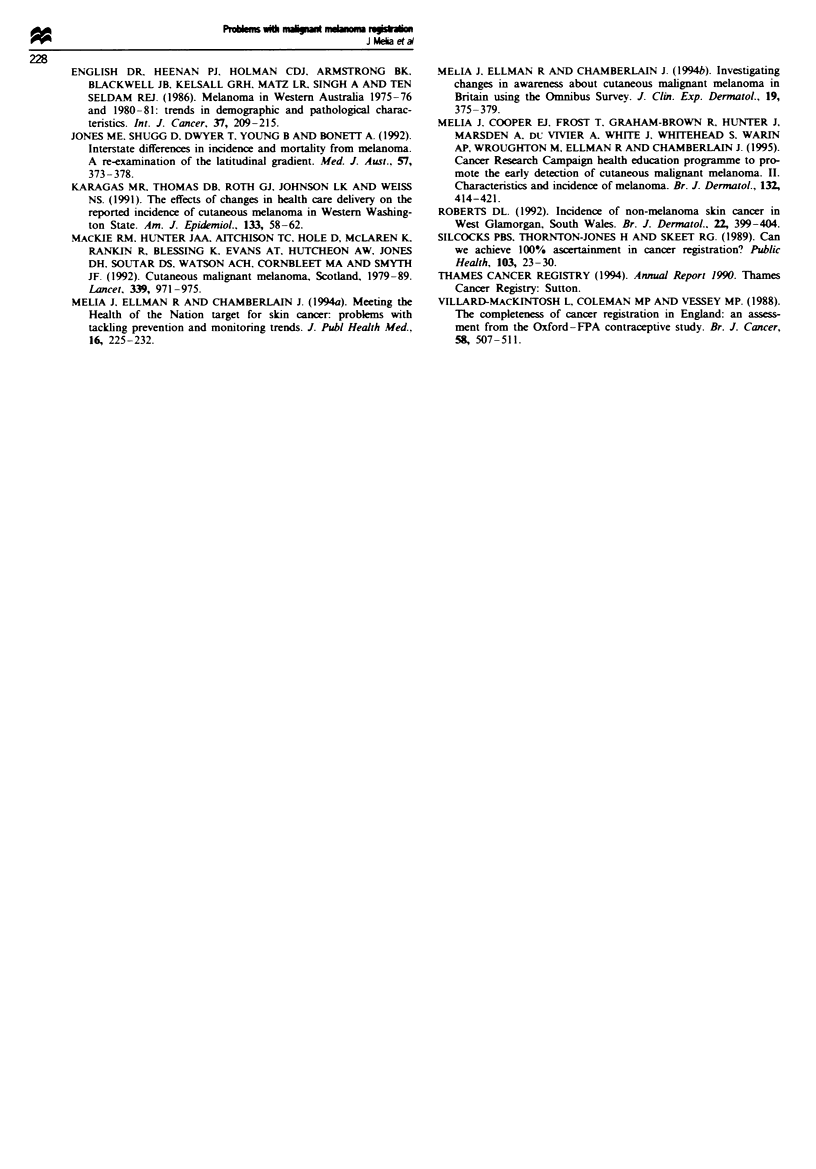# Problems with registration of cutaneous malignant melanoma in England.

**DOI:** 10.1038/bjc.1995.307

**Published:** 1995-07

**Authors:** J. Melia, T. Frost, R. Graham-Brown, J. Hunter, A. Marsden, A. du Vivier, A. P. Warin, J. White, S. Whitehead, M. Wroughton

**Affiliations:** Cancer Screening Evaluation Unit, Institute of Cancer Research, Sutton, Surrey, UK.

## Abstract

The aim of the study was to assess the completeness and accuracy of cancer registration for cutaneous malignant melanoma. The study was conducted in seven health districts in England and one health board in Scotland from 1987 to 1989 with a total resident population of 3.6 million. Records from pigmented lesion clinics and pathology laboratories collected during the Cancer Research Campaign's health education programme to promote the early detection of melanoma were matched with cancer registrations from a total of five regional cancer registries. In England 74% out of a total of 642 cases of invasive malignant melanomas (ICD 172) and 44% out of a total of 155 in situ melanomas (ICD 232) had been registered compared with 96% and 100% respectively in Scotland. A significantly higher proportion of late-stage cases was found among registered than among non-registered cases in England (P < 0.001). In all registries the majority of superficial spreading in situ melanomas were miscoded as invasive cases. The annual incidence of invasive malignant melanoma in the English study areas was found to be seven per 100,000 in men and 11 per 100,000 in women, similar to that reported in Scotland. The registries are best at recording thick or late-stage melanomas. As the skin cancer target for Health of the Nation depends on monitoring trends in the incidence of malignant melanoma, future improved ascertainment of cases and changes in the type of cases being registered must be taken into account.


British Journal of Cancer (1995) 72, 224-228

© 1995 Stockton Press All rights reserved 0007-0920/95 $12.00

### Problems with registration of cutaneous malignant melanoma in England

J Melia[1], T Frost[2], R Graham-Brown[3], J Hunter[4], A Marsden[5], A du Vivier[6], AP Warin[2], J White[7], S Whitehead[8], M Wroughton[9], R Ellman[1] and J Chamberlain[1]

*[1]Cancer Screening Evaluation Unit, Institute of Cancer Research, Section of Epidemiology, D Block, Cotswold Road, Sutton, Surrey SM2 5NG; [2]Department of Dermatology, Royal Devon & Exeter Hospital, Barrack Road, Exeter EX2 5DE; [3]Department of Dermatology, Leicester Royal Infirmary, Infirmary Square, Leicester LE1 5WW; [4]Department of Dermatology, Level 4, Phase 1, The Royal Infirmary, Edinburgh EH3 9YW; [5]Department of Dermatology, St. George's Hospital, Blackshaw Road, London SW17 0QT; [6]Department of Dermatology, King's College Hospital, Denmark Hill, London SE5 9RS; [7]Royal South Hants Hospital, Graham Road, Southampton, Hants SO9 4PE; [8]Department of Public Health Medicine, South Derbyshire HA, Boden House, Main Centre, Derby DE1 2PH; [9]Department of Dermatology, Queen's Medical Centre, Nottingham NG7 2UH, UK.*

**Summary** The aim of the study was to assess the completeness and accuracy of cancer registration for cutaneous malignant melanoma. The study was conducted in seven health districts in England and one health board in Scotland from 1987 to 1989 with a total resident population of 3.6 million. Records from pigmented lesion clinics and pathology laboratories collected during the Cancer Research Campaign's health education programme to promote the early detection of melanoma were matched with cancer registrations from a total of five regional cancer registries. In England 74% out of a total of 642 cases of invasive malignant melanomas (ICD 172) and 44% out of a total of 155 *in situ* melanomas (ICD 232) had been registered compared with 96% and 100% respectively in Scotland. A significantly higher proportion of late-stage cases was found among registered than among non-registered cases in England ($P < 0.001$). In all registries the majority of superficial spreading *in situ* melanomas were miscoded as invasive cases. The annual incidence of invasive malignant melanoma in the English study areas was found to be seven per 100 000 in men and 11 per 100 000 in women, similar to that reported in Scotland. The registries are best at recording thick or late-stage melanomas. As the skin cancer target for Health of the Nation depends on monitoring trends in the incidence of malignant melanoma, future improved ascertainment of cases and changes in the type of cases being registered must be taken into account.

**Keywords**: cutaneous melanoma; cancer registration

In the government White Paper 'Health of the Nation' a target for skin cancer has been set 'to halt the year-on-year increase in the incidence of skin cancer by 2005' (Department of Health, 1992*a*). Monitoring trends in the incidence of non-melanocytic skin cancers (International Classification of Diseases, 9th revision: ICD9) is known to be problematic because of incomplete registration (Roberts, 1992). In 1993 some English registries ceased registration of basal cell carcinomas.

Although there has undoubtedly been a true increase in the incidence of malignant melanoma (ICD9 172) in England in recent decades, some of the steep rise is likely to be explained by improved ascertainment of cases for registration. Of 20 cases of melanoma occurring from 1968 to 1985 in an English population of 17 000 women aged 25-49 years, eight (40%) had not been registered (Villard-Mackintosh *et al*., 1988). More recently the Thames Cancer Registry (1994) estimated that 28-31% of malignant melanomas had not been registered, using a calculation based on the distribution of survival, cause of death and chance of being registered from medical records. In contrast, Scotland is believed to have nearly complete registration because of the Scottish Melanoma Group register established in 1979 (MacKie *et al*., 1992); this register, set up by dermatologists, surgeons and pathologists, exchanges information every year with the Scottish Cancer Registries. The higher incidence rates reported in Scotland (7.1 per 100 000 and 10.4 per 100 000 in males and females respectively) than in England (3.9 per 100 000 and 6.1 per 100 000 in males and females respectively) (Melia *et al*., 1994*a*) for 1986-88 standardised by age to the 1987 England and Wales population may in part have occurred because of more complete registration in Scotland.

A study had been set up in England and Scotland to evaluate the long-term effects of a health education programme for the early detection of melanoma (Ellman, 1991). The opportunity was taken to use the data to investigate the extent of under-registration of malignant melanoma, biases arising in the type of cases being registered and errors in diagnostic coding. The Cancer Research Campaign (CRC) funded a health education programme from 1987 to 1989 in seven English health districts and one Scottish health board. To study the impact of the programme on the incidence of malignant melanoma, data were collected from pigmented lesion clinics, pathology laboratories and cancer registries. The data from these sources have been compared to investigate the registration of melanoma.

### Methods

### *Data collection*

The aim of the health education programme was to promote the early detection of melanoma in the general public. Seven district health authorities in England (Camberwell, Exeter, Leicester, Nottingham, Southampton and South-West Hampshire, and Wandsworth, Merton and Sutton combined) and Edinburgh City within Lothian Health Board were selected because local melanoma registries were already being run by the dermatology clinics. The areas yielded a target population of 3.6 million from a range of rural and urban areas. The CRC funded pigmented lesion clinics (PLCs) in each area to cope with the extra workload expected after the launch of the programme on 8 July 1987. A standardised form was used at the PLCs to collect data on the demographic and clinical details of each patient. The programme, which promoted its message through the local media and leaflets and posters, was most active during the summer of 1987, but additional publicity was promoted in some study areas in 1988 and 1989. The PLCs were funded until the end of 1989.

The main aim of the evaluation study is to investigate the

Correspondence: J Melia
Received 16 September 1994; revised 30 January 1995; accepted 20 February 1995

effect of the campaign on mortality from melanoma. To establish a database for this investigation, information on melanoma cases was collected from four main sources: the PLCs; local melanoma registers based on patients attending dermatology departments in hospital rather than population based; the main histopathology laboratories serving the study areas; and regional cancer registries. It was impractical in this study to contact all histopathology laboratories which might have diagnosed patients treated outside but resident in the study areas, especially in the Thames regions, where there is considerable cross-boundary flow for treatment. Although most laboratories have computerised databases, many did not record an address, which is essential to identify people according to area of residence, and many could not download information on disk. As a result the histopathological data which were used in the study had to be extracted manually.

*Definition of cases*

In this paper completeness of registration is estimated from the proportion of registered cases among all cases of invasive malignant melanoma and *in situ* melanoma of the skin, first diagnosed during the campaign period 8 July 1987 to 31 December 1989. Malignant melanomas of the eye and urogenital area have been excluded. A case is defined as a person counted at the time of the first melanoma being diagnosed. Synchronous melanomas are counted as one case and subsequent cases or recurrences have not been included in the analyses.

Cases of invasive malignant melanoma recorded by PLCs, pathology laboratories and local hospital-based registers included superficial spreading, lentigo maligna melanomas, acral, nodular and non-specified types of malignant melanoma. *In situ* melanomas included cases of superficial spreading melanoma *in situ*, lentigo maligna and non-specified types of malignant melanoma recorded as *in situ* or with a Breslow thickness of zero. All cases from these sources were pathologically confirmed. From the cancer registries, cases diagnosed during the study period which were registered by March 1994 were included. The proportion of registered melanomas recorded as pathologically confirmed ranged from 89% at one English registry to 99% in Scotland.

Invasive malignant melanoma is coded as ICD 172 with a behaviour code ending with 3 using the 9th revision of the International Classification of Diseases (ICD9), or alternatively as ICD 173 with behaviour code ending with a 3 and morphology within the range 872–879 when using the specialty-based adaptation of the 9th revision for oncology (ICDO). *In situ* melanomas should be coded as ICD 172 and behaviour code ending with 2 when using the 9th revision or as ICD 173 and behaviour code ending with 2 when using ICDO. The data collected from other sources in the CRC study also used Breslow thickness to help define invasive and *in situ* lesions, lesions with a Breslow thickness of zero or not applicable being coded as *in situ*. This may not always happen for cases collected by the registries, and some may depend solely on whether or not the term *in situ* is clearly specified in the patient's notes.

Where registered cases were matched to the cases identified from other sources, and discrepancies found, the clinical and pathology records were checked to confirm the correct diagnosis. These showed that the data from other sources were correct for 99% of cases and therefore, in the analyses of registration rates and characteristics of registered cases, priority was given to the diagnosis recorded from other sources, not the cancer registries. For cases recorded only by the registry, it was not feasible to check the pathology records because of the large number of laboratories involved, so the diagnosis recorded by the registry was assumed to be correct in the analyses.

*Analysis*

By matching cases identified from other sources with those on the cancer register, the proportions of invasive malignant melanoma and *in situ* melanomas which had been correctly registered were found. If data on Breslow thickness were missing, cases with metastases or recorded as late stage at time of diagnosis were classified in a group which included cases where Breslow thickness was $>3.0$ mm. For registered cases a comparison of the diagnosis as recorded in hospital case notes and by the cancer registries was made. The analyses were conducted using simple tabulations and the chi-squared test to test for significant differences between groups. Multifactorial regression analyses were conducted to study the relationship between registration and age, sex and Breslow thickness using the SPSS computer package.

### Results

A total of 787 invasive malignant melanomas and 179 *in situ* melanomas were diagnosed in the study areas during the study period. For the invasive malignant melanomas, a significantly lower proportion of cases diagnosed in England were registered (74%) than among cases diagnosed in Scotland (96%) ($P<0.001$). In the four English registries the ascertainment rate ranged from 66% to 88% (Table I). Analysis of invasive malignant melanoma by source showed that overall 173 (22%) cases were identified by the registries alone, 172 (22%) cases by other sources alone and 442 (56%) cases by both. The proportion of cases identified by the registry alone was highest in London (37%), where there is cross-boundary flow of treatment and cases had been treated elsewhere.

*In situ* melanomas were substantially under-registered in the English areas (44%, range 23–58%) compared with 100% registration in Scotland (Table I). Nineteen (10%) cases in England and Scotland (all lentigo malignae) were found on the registries alone, 87 (49%) by other sources alone and 73 (41%) by both.

In England, the percentage of invasive cases registered was higher for thick melanomas ($\geqslant 3.1$ mm) than thin melanomas ($\leqslant 1.5$ mm) (Table II, $P<0.001$). The distribution of non-registered and registered cases also differed by age, there being higher proportions of non-registered cases in the younger than other age groups ($P<0.01$). However, age was related to the distribution of Breslow thickness, with younger age groups having significantly thinner Breslow thicknesses. There was no significant variation in registration according to sex. In multifactorial regression analyses to study the relation between registration, age, sex and Breslow thickness, Breslow thickness was the only variable significantly related to registration ($P<0.01$).

In Edinburgh only six cases of invasive malignant melanoma had not been registered. Two had the diagnosis misclassified in the registry, one person had a dual address in Edinburgh and London and three had not been reported by the registry.

Looking only at cases recorded by both the registries and other sources, the diagnosis recorded from hospital records was compared with the cancer registry codings. For the 442 cases recorded as invasive malignant melanoma by other sources, there was 99% agreement in the diagnoses between the registries and other sources. For the 73 cases recorded as *in situ* melanoma by the other sources, there was a consistent discrepancy across all registries in England and Scotland. Lentigo maligna was correctly coded by the registries as *in situ* melanoma for 25 out of 27 cases. However, 33 out of 37 cases of *in situ* superficial spreading melanoma and a further nine cases of unspecified melanoma *in situ* were recorded as invasive malignant melanoma by the registries.

Taking all cases of invasive malignant melanoma identified from all sources, the annual incidence rate of melanoma is estimated to be seven and 11 per 100 000 in males and females respectively in the English study areas and nine and 15 per 100 000 respectively in Edinburgh, age standardised to the population of England and Wales in 1988. Taking registered cases only, the incidence rate of malignant melanoma in the study areas is very similar to that reported

**Table I** Percentage (number in brackets) of invasive malignant melanomas and *in situ* melanomas diagnosed between 1987 and 1989 according to data source[b]

| | England | | | | | Scotland | |
|---|---|---|---|---|---|---|---|
| *Study area* | *Exeter* | *Camberwell, Merton, Sutton and Wandsworth* | *Leicester and Nottingham* | *Southampton and S.W. Hampshire* | *Total* | *Edinburgh City* | *Total* |
| Region of registry | South Western | Thames | Trent | Wessex | | Scotland | |
| *Invasive malignant melanoma ICD9 172* | | | | | | | |
| Total number of cases ascertained from all sources | 100 (108) | 100 (139) | 100 (263) | 100 (132) | 100 (642) | 100 (145) | 100 (787) |
| Per cent registry only[a] | 13 (14) | 37 (52) | 12 (32) | 30 (40) | 21 (138) | 24 (35) | 22 (173) |
| Per cent both registry and non-registry sources[a] | 53 (57) | 31 (42) | 62 (162) | 58 (77) | 53 (338) | 72 (104) | 56 (442) |
| Per cent other sources only | 34 (37) | 32 (45) | 26 (69) | 12 (15) | 26 (166) | 4 (6) | 22 (172) |
| In situ *melanoma ICD9 232* | | | | | | | |
| Total number of cases ascertained from all sources | 100 (44) | 100 (21) | 100 (78) | 100 (27) | 100 (155) | 100 (24) | 100 (179) |
| Per cent registry only[a] | 2 (1) | – | 9 (7) | 2 (17) | 6 (10) | 37 (9) | 10 (19) |
| Per cent both registry and non-registry sources[a] | 21 (9) | 48 (10) | 44 (34) | 41 (5) | 38 (58) | 63 (15) | 41 (73) |
| Per cent other sources only | 77 (34) | 52 (11) | 47 (37) | 42 (5) | 56 (87) | – | 49 (87) |

[a]Registered by March 1994. [b]All cases pathologically confirmed except a small proportion of cases identified by the registries alone as explained in Methods.

**Table II** The percentage (number given in brackets) of registered and non-registered cases of invasive malignant melanoma in the English study areas according the Breslow thickness

| | Breslow thickness (mm)[a] | | | | Breslow thickness |
|---|---|---|---|---|---|
| | $\leqslant 1.5$ | 1.51–3.0 | $\geqslant 3.1$[b] | Total | not known |
| Registered cases | 65.7 (199) | 67.5 (77) | 84.8 (95) | 70.1 (371) | 105 |
| Non-registered cases | 34.3 (104) | 32.5 (37) | 15.2 (17) | 29.9 (158) | 8 |
| Total | 100 (303) | 100 (114) | 100 (112) | 100 (529) | 113 |

[a]$\chi^2$ with two d.f. = 14.8 for difference in registration by Breslow thickness ($P<0.001$). [b]Includes registered cases with no data on Breslow thickness but recorded with late stage or metastases.

by the cancer registries for the whole of England (six and nine per 100 000 in males and females respectively) and the whole of Scotland (nine and 12 per 100 000 for males and females respectively).

### Discussion

The under-registration of malignant melanoma identified in this study has implications for the Health of the Nation target for skin cancer (Department of Health, 1992*a*). It has been proposed in the Specification of National Indicators for the Health of the Nation (Department of Health, 1992*b*) that incidence should be monitored separately for both non-melanocytic skin cancers (ICD9 173) and malignant melanoma (ICD9 172). Both clearly suffer from under-registration, and incidence rates can be expected to show an apparent increase in the next few years if ascertainment by the cancer registries improves and if there is increased diagnosis of early-stage, sometimes non-progressive, disease. The cancer registries seem best at recording late-stage malignant melanomas, and therefore ideally the incidence rate of malignant melanoma should be monitored by stage using an index such as Breslow thickness. Breslow thickness has a strong association with prognosis with only 25% of patients with tumours thicker than 3.0 mm surviving 5 years compared with 91% of patients with tumours less than 1.51 mm (Breslow, 1970).

Under-registration of invasive malignant melanoma was low in this study (25%) compared with the 40% reported by Villard-Mackintosh *et al.* (1988). Comparisons between the two studies must be made with caution because ascertainment of cases will vary between registries and over time, and also the present study had a much larger population. This study reports on melanomas diagnosed from 1987 to 1989 for the total population in areas covered by four English registries, whereas the other study focused on women aged 25–49 years reporting melanomas from 1968 to 1985. An alternative comparison with the Thames Cancer Registry's own estimate of under-ascertainment for malignant melanoma showed very similar results to those in the CRC study (Thames Cancer Registry, 1994). Variation in under-registration between registries in part reflects the different methods of data collection used by the registries. Among the English registries registration was highest for the area covered by the Wessex registry, which has established good links with pathology laboratories for several years.

There are two limitations with the data set which may have led to underestimates of the extent of under-ascertainment. First, it is possible that ascertainment was more complete in the CRC study areas than elsewhere because local dermatological melanoma registers, activities of the health education programme and associated research may have encouraged exchange of information between the hospitals and cancer registries. Second, as it was impractical to collect data from all pathology laboratories diagnosing cases treated outside the study areas, some melanomas may have remained undetected, particularly in areas where pathology links with registries were poor. Thus the ascertainment rate overall in England may be lower than that estimated in this study. On the other hand, the presence of local registers and research can sometimes hinder cancer registration if, for example, patients' notes are removed for these purposes and thus cannot be found in the routine searches by registry staff.

Under-ascertainment is bound to vary between registries across England, and the four English registries may not be representative of all registries. However, the registries had a mix of data collection methods similar to those used elsewhere. Further analysis of the data with respect to stage and patient characteristics (Melia *et al.*, 1995) has shown similarities between the full data set for the study areas and that for the whole of Scotland (MacKie *et al.*, 1992) where there is near complete registration suggesting that there is no substantial bias in the data set presented here.

The under-registration of cancers in England and Wales and various reasons for this are well recognised (Benn *et al.*, 1982; Silcocks *et al.*, 1989). The important issues arising from the present study are firstly the biases in types of melanomas being registered, which will affect the interpretation of trends in incidence, and secondly errors in the diagnostic coding of *in situ* cases.

The main bias is the incomplete registration of thin malignant melanomas. As awareness about melanoma increases in the general public in Britain, the number of thin malignant melanomas at diagnosis will also increase (MacKie *et al.*, 1992; Melia *et al.*, 1994*b*). Most cases of thin malignant melanoma are treated on an out-patient basis with simple excision, require no further treatment and rarely lead to death. Therefore under-registration of these cases will be high in registries which depend largely on data from in-patient discharge records, radiotherapy departments and death certificates. Therefore pathology laboratories are a very important source.

Both the under-registration and bias in type of cases being registered could lead to misinterpretation of geographical variation in incidence rates for malignant melanoma both within the UK and abroad. Ascertainment of melanoma cases by registries is believed to be high in areas of Australia where pathology records are used as a source of information (Bonett *et al.*, 1989). There has certainly been an increase in the incidence of malignant melanoma in Australia, but some of the rise in parts of Australia may be explained by improved ascertainment (Jones *et al.*, 1992). In contrast, in one state in USA the under-registration of melanoma cases increased from 2% in 1974 to 21% in 1984 (Karagas *et al.*, 1991), probably because of increased diagnosis of thin lesions which were not being picked up by the registries.

The problem with the coding of *in situ* superficial spreading melanomas as invasive may have occurred because of lack of understanding of the use of the matrix system for the coding of such lesions in ICD9 and ICDO. Some confusion may also have been caused by a variety of terms used in clinical records. Thus, *in situ* superficial spreading melanomas may not have been recognised as such if the term 'malignant' was used or if '*in situ*' was not clearly specified. Although it will have led to an over-reporting of invasive malignant melanoma, the numbers are too small to have a major impact on estimates of incidence. It is possible that with increased reporting of thin melanomas this could become a sizeable problem. It has also been reported in Australia as a source of error (English *et al.*, 1986), and a similar coding error has been reported for cervical cancer (Choyce and McAvoy, 1990). Special studies would help to clarify the extent of this problem for other cancer sites.

In England ascertainment of many cancers will improve as the registries develop links with pathology laboratories (Codling *et al.*, 1990). The registries should also be encouraged to record prognostic indices for melanoma as trends in melanomas with a poor prognosis will precede changes in mortality and they are an important marker for improvements in early detection of the disease. Breslow thickness is one of the most reliable measures of prognosis, although there are some problems with its accuracy (Colloby *et al.*, 1991). The vertical growth component, which is positively correlated with Breslow thickness, may be a better indicator of prognosis as it requires assessment of two additional components: cytology of the cells and more importantly, the level of invasion of the dermal microenvironment (Clark *et al.*, 1989). Completeness of these data will depend on both the pathologists and registry staff. The proportion of invasive malignant melanomas for which Breslow depth is routinely measured in England is not known. The completeness of these data (95%) at the pathology laboratories in this study may be higher than that occurring elsewhere if there is no special interest in melanoma. The completeness of data recorded by registry staff does need to be improved as at one registry in this study only 7% of Breslow thicknesses given in the hospital notes had been recorded by the registry.

The main factor affecting the interpretation of trends in incidence of malignant melanoma in England is the underestimate of incidence in young adults and those with thin tumours. As ascertainment continues to improve, some of the apparent increase in incidence seen in the future will reflect this improvement. Incidence rates for malignant melanoma should ideally be monitored by Breslow thickness or stage as registries have been best at recording late-stage cancers and most improvement in ascertainment is expected for early-stage thin cancers. Increased collaboration between pathology laboratories, dermatologists and cancer registries will help to resolve problems with registering melanoma and increasing the recording of data on Breslow thickness.

**Acknowledgements**
We thank the Cancer Research Campaign (CRC) for funding this research and the staff at the DH Cancer Screening Evaluation Unit for help with data processing. We also thank all those working in the pigmented lesion clinics, pathology laboratories, Scottish Melanoma Group and the cancer registries for Scotland and the South Western, Thames, Trent and Wessex Regions for data collection.

**References**

BENN RT, LECK I AND NWENE UP. (1982). Estimation of completeness of cancer registration. *Int. J. Epidemiol.*, **11**, 362–367.

BONETT A, RODER D AND ESTERMAN A. (1989). Epidemiological features of melanoma in South Australia: implications for cancer control. *Med. J. Aust.*, **151**, 502–509.

BRESLOW A. (1970). Cross-sectional area and depth of invasion in the prognosis of cutaneous melanoma. *Ann. Surg.*, **172**, 902–908.

CHOYCE A AND MCAVOY BR. (1990). Cervical cancer screening and registration – are they working? *J. Epidemiol. Commun. Hlth.*, **44**, 52–54.

CLARK WHJ ELDER DE, GUERRY IV D, BRAITMAN LE, TROCK BJ, SCHULTZ D, SYNNESTVEDT M AND HALPERN AC. (1989). Model predicting survival in Stage I melanoma based on tumour progression. *J. Natl Cancer Inst.*, **81**, 1893–1904.

CODLING BW, PHEBY D, HAGEN DL AND DUFFIN MF. (1990). Cancer registration by linking pathology and district PAS data. *Br. J. Cancer*, **62**, 271–274.

COLLOBY PS, WEST KP AND FLETCHER A. (1991). Observer variation in the measurement of Breslow depth and Clark's level in thin cutaneous malignant melanoma. *J. Pathol.*, **163**, 245–250.

DEPARTMENT OF HEALTH (1992*a*). *The Health of the Nation: A Strategy for Health in England*. Department of Health, HMSO: London.

DEPARTMENT OF HEALTH (1992*b*). *The Health of the Nation: Specification of National Indicators*. Department of Health, HMSO: London.

ELLMAN R. (1991). Screening for melanoma in the UK. In *Cancer Screening*. UICC Project on Evaluation of Screening for Cancer. Miller AB, Chamberlain J, Day NE, Hakama M, Prorok PC, (eds) pp. 257–266. Cambridge University Press: Cambridge.

ENGLISH DR, HEENAN PJ, HOLMAN CDJ, ARMSTRONG BK, BLACKWELL JB, KELSALL GRH, MATZ LR, SINGH A AND TEN SELDAM REJ. (1986). Melanoma in Western Australia 1975-76 and 1980-81: trends in demographic and pathological characteristics. *Int. J. Cancer*, **37**, 209-215.

JONES ME, SHUGG D, DWYER T, YOUNG B AND BONETT A. (1992). Interstate differences in incidence and mortality from melanoma. A re-examination of the latitudinal gradient. *Med. J. Aust.*, **57**, 373-378.

KARAGAS MR, THOMAS DB, ROTH GJ, JOHNSON LK AND WEISS NS. (1991). The effects of changes in health care delivery on the reported incidence of cutaneous melanoma in Western Washington State. *Am. J. Epidemiol.*, **133**, 58-62.

MACKIE RM, HUNTER JAA, AITCHISON TC, HOLE D, McLAREN K, RANKIN R, BLESSING K, EVANS AT, HUTCHEON AW, JONES DH, SOUTAR DS, WATSON ACH, CORNBLEET MA AND SMYTH JF. (1992). Cutaneous malignant melanoma, Scotland, 1979-89. *Lancet*, **339**, 971-975.

MELIA J, ELLMAN R AND CHAMBERLAIN J. (1994*a*). Meeting the Health of the Nation target for skin cancer: problems with tackling prevention and monitoring trends. *J. Publ Health Med.*, **16**, 225-232.

MELIA J, ELLMAN R AND CHAMBERLAIN J. (1994*b*). Investigating changes in awareness about cutaneous malignant melanoma in Britain using the Omnibus Survey. *J. Clin. Exp. Dermatol.*, **19**, 375-379.

MELIA J, COOPER EJ, FROST T, GRAHAM-BROWN R, HUNTER J, MARSDEN A, DU VIVIER A, WHITE J, WHITEHEAD S, WARIN AP, WROUGHTON M, ELLMAN R AND CHAMBERLAIN J. (1995). Cancer Research Campaign health education programme to promote the early detection of cutaneous malignant melanoma. II. Characteristics and incidence of melanoma. *Br. J. Dermatol.*, **132**, 414-421.

ROBERTS DL. (1992). Incidence of non-melanoma skin cancer in West Glamorgan, South Wales. *Br. J. Dermatol.*, **22**, 399-404.

SILCOCKS PBS, THORNTON-JONES H AND SKEET RG. (1989). Can we achieve 100% ascertainment in cancer registration? *Public Health*, **103**, 23-30.

THAMES CANCER REGISTRY (1994). *Annual Report 1990*. Thames Cancer Registry: Sutton.

VILLARD-MACKINTOSH L, COLEMAN MP AND VESSEY MP. (1988). The completeness of cancer registration in England: an assessment from the Oxford-FPA contraceptive study. *Br. J. Cancer*, **58**, 507-511.